# Further insight into AE37 peptide vaccination in prostate cancer

**DOI:** 10.4155/fsoa-2017-0005

**Published:** 2017-05-11

**Authors:** Eleftheria A Anastasopoulou, Ioannis F Voutsas

**Affiliations:** 1 Cancer Immunology & Immunotherapy Center, Saint Savas Cancer Hospital, Athens, Greece

**Keywords:** cancer vaccines, clinical trials, immunotherapy, patient selection, pre-existing immunity, prostate cancer

Cancer peptide vaccination, as an immunotherapeutic approach against solid tumors, is currently employed in several clinical research protocols. The underlying mechanism of peptide-based vaccines involves the generation of a T-cell immune response against tumor or enhancement of an endogenous antitumor immunity pre-existing in the host [1]. Although the rationale of cancer vaccination studies seems to be promising, therapeutic efficacy is rather limited [2,3]. One reason that could account for that is the inappropriate clinical trial design and patient selection, rather than the vaccine itself [3]. It is important to underline that the antitumor immune response is influenced by the respective immunological status and tumor-cell characteristics, thus implying heterogeneity among patients [4]. In respect to this, T-cell responses against certain peptides, including those derived from tumors, are mediated by specific human leukocyte antigen (HLA) molecules, therefore, the HLA polymorphism is another factor reflecting this variation in patients’ immunological response [5]. Given that, patients’ selection in vaccination studies requires careful consideration, so as to achieve therapeutic efficacy.

According to recent reports, pre-existing host immunity is essential in order to gain a therapeutic benefit, in the context of peptide cancer vaccines [2,6]. More specifically, patients with a pre-existing immunity to the vaccine antigen can develop fast and robust immune responses, upon vaccination. In addition, since pre-existing immunity against peptides integrated in the vaccine formulation, has been shown to be a predictive biomarker of response and therapeutic benefit, it should be used to select patients who are likely to respond to the vaccine [7]. On the contrary, delayed and insufficient immune responses are observed in patients with no immunological memory to the vaccine antigen and especially those with advanced cancer, characterized also by high immunosuppresion levels due to disease progression [8].

Considering the heterogeneity among individuals along with the role of pre-existing immunity prior to vaccination, a more personalized approach in peptide-based vaccination studies could result in better responses and further contribute to the development of effective cancer vaccines. In line with this, the HLA phenotype and levels of pre-existing immunity could be employed in clinical trials in order to identify cancer patients that could benefit from a specific peptide vaccine. In this respect, a recent study from our group showed that prostate cancer patients vaccinated with the Ii-key hybrid HER2 peptide [9], exhibit longer progression-free survival when they have increased levels of pre-existing immunity to the respective native HER2 peptide (AE36). In this study, additionally to enzyme-linked immunosorbent spot IFN-γ, we used the local reaction (LR) at the inoculation site of the first vaccine (500 μg peptide + 125 μg granulocyte-macrophage colony stimulating factor) (LR1) as an approach for assessing pre-existing immunity, besides the generally accredited immunomonitoring method of delayed type hypersensitivity (DTH) [10]. In particular, in standard DTH reactions (100 μg AE36 or AE37 without granulocyte-macrophage colony stimulating factor) used in the protocol prevaccination, was not found to be sufficient to evaluate pre-existing immunity for HER2, given the low concentration of peptide and the absence of immunoadjuvant, to trigger a usually low immunological memory response to self-antigens. Therefore, we considered LR1 as a more appropriate method to evaluate HER2 pre-existing immunity, in the context of immunomonitoring, which is also in agreement with previous reports describing that LR to the vaccination site can be used to evaluate immune responses [11].

Bearing in mind that AE37 is actually a multiepitope vaccine, potent to stimulate both CD4^+^ and CD8^+^ T cells, aiming at the induction of a generalized immune response [1], we decided to investigate the levels of pre-existing immunity to the vaccine. Most importantly, given the fact that the epitope-spreading effect is a significant parameter usually assessed in the context of vaccine evaluation, we investigated specific immunity to MHC class I restricted epitopes not integrated in the vaccine formulation, using specific multimers (dextramers) [12]. In particular, we assessed HLA-A2 and -A24^+^ patients, since these were the most commonly expressed alleles in our study group. More specifically, we investigated specific CD8 T cells against tumor-associated epitopes derived from HER2 (HER2_85–94_ [HER2_85_], HER2_435–443_ [HER2_435_], HER2_369–377_[E75]), PSA (PSA_146–154_[PSA_146_], PSA_153–161_[PSA_153_]), hTERT (hTERT_540–548_), PSMA (PSMA_27–35_) and survivin (survivin M2_96–104_ [SURV_96_], survivin_20–28_ [SURV_20_]). Our findings showed that AE37 vaccinations boost specific antitumor immune responses against epitopes not integrated in the vaccine formulation, further implying an epitope-spreading effect. Namely, we detected relatively high frequencies of specific CD8^+^ T cell pre-existing immunity against PSA_153–161_, an HLA-A24-restricted epitope, which was further enhanced upon vaccination and was associated with favorable clinical outcome in the respective HLA carriers. Moreover, we detected high pre-existing frequencies of specific CD8^+^ T cells for E75 (HER2_369–377_) and PSA_146–151_ in HLA-A2^+^ patients that were also enhanced upon AE37 vaccination. However, specific E75 CD8^+^ T-cell immunity in HLA-A2^+^ patients, either pre-existing or AE37 induced, seems to correlate with an unfavorable clinical outcome.

The immunological status of each patient along with the shifting tumor profile might explain why cytotoxic T lymphocyte E75-specific immunity failed to be interpreted into an effective clinical response. It is worth mentioning that under the pressure of the immune system, tumors alter the expression of HLA class I molecules, from total loss to reduced expression, a common escape mechanism that allows the selective growth of less immunogenic tumor variants during immunoediting [13]. In line with this, it has been previously described that high HER2 expressing tumors are characterized by impaired antigen presentation, resulting in poor recognition of the respective tumor antigen by specific tumor-reactive CD8^+^ T cells [14]. Beside this, preclinical studies have noted the role of HER2 in the downregulation of HLA class I molecules, while results from clinical trials suggest that E75 vaccination might not favor breast cancer patients with tumors expressing high levels of HER2 [15]. In support to our results, a recent study by Tran *et al.* [16] reported that E75-based peptide vaccination can inhibit tumor growth only in low HER2 expressing tumors in HLA-A2^+^ transgenic mice. This and the fact that HER2 expression is known to increase significantly following disease progression in prostate cancer [17], could account for the inability of E75-specific cytotoxic T lymphocytes, detected in our patients, to exert an effective antitumor immune response with direct clinical impact. In other words, this potential downregulation of the HLA-A2 in high HER2 expressing tumors, could explain the unfavorable clinical outcome (shorter progression-free survival) observed in our patients exhibiting high pre-existing immunity against E75 or with induced E75 immunity upon vaccination with AE37.

To conclude, our data, although hypothesis generating, might pave the way to unravel the prognostic/predictive significance of pre-existing antitumor immunological memory and how it can be employed so as to improve immunotherapeutic strategies. Recent cancer vaccination modalities are adopting a more personalized approach since they are based on the identification of patient’s tumor mutanome and the prediction of potential neoepitopes that could represent attractive tumor antigens [18]. It is noteworthy to mention that this personalized approach is also reflected by the development of personalized peptide vaccines (PPV), which may include a maximum of four HLA-matched peptides [2], based on the pre-existing host immunity before vaccination. Results from Phase I and II clinical trials, have shown that PPV can induce specific immune responses with promising clinical outcomes in castrate-resistant prostate cancer patients [19]. Hence, considering that in the last few years several clinical studies have employed PPV for the treatment of the prostate cancer in HLA-A24^+^ and -A2^+^ patients [2,19,20], our findings could further contribute to the development of the respective immunotherapeutic approach. In this respect, future immunotherapeutic protocols with the AE37 vaccine, could possibly achieve better clinical responses by recruiting patients with a pre-existing immunity to the native peptide. Regarding patient selection, we recommend that a DTH test with higher antigen concentration, with or without the immunoadjuvant, could possibly be used to successfully evaluate the levels of pre-existing immunity in patients prior to vaccination, along with the *in vitro* assessment of IFN-γ enzyme-linked immunosorbent spot.

Considering all the above, it should be noted that employing CD8^+^ T-cell epitopes in peptide-based vaccines seems to be rather challenging, given the impaired expression of HLA class I molecules in tumor cells, thus research in this direction should remain viable.
